# The effect of connective tissue graft or a collagen matrix on epithelial differentiation around teeth and implants: a preclinical study in minipigs

**DOI:** 10.1007/s00784-023-05080-5

**Published:** 2023-06-10

**Authors:** Alexandra Stähli, László Párkányi, Sofia Aroca, Andreas Stavropoulos, Frank Schwarz, Anton Sculean, Dieter D. Bosshardt

**Affiliations:** 1 Department of Periodontology, School of Dental Medicine, University of Bern, Freiburgstrasse 7, 3010 Bern, Switzerland; 2 Department of Periodontology, Faculty of Dentistry, University of Szeged, 6720 Tisza Lajos Körút 64, Szeged, Hungary; 3 Department of Periodontology, Faculty of Odontology, Malmö University, Carl Gustafs Väg 34, 20506 Malmo, Sweden; 4 Department of Oral Surgery and Implantology, Goethe University, Theodor-Stern-Kai 7, 60596 Frankfurt, Germany; 5 Robert K. Schenk Laboratory of Oral Histology, School of Dental Medicine, University of Bern, Freiburgstrasse 3, 3010 Bern, Switzerland

**Keywords:** Connective tissue graft, Keratinization, Minipig, Single tooth, Dental implants

## Abstract

**Objectives:**

This study aimed to histologically evaluate the healing at 8 weeks after coronally advanced flap (CAF) with either a superficial (SCTG) or deep palatal connective tissue graft (DCTG), or a collagen matrix (CM) to cover recession defects at teeth and implants.

**Material and methods:**

One mandibular side of 6 miniature pigs received each 3 titanium implants 12 weeks after extraction. Eight weeks later, recession defects were created around implants and contralateral premolars and 4 weeks later randomly subjected to CAF + SCTG, CAF + DCTG, or CAF + CM. After 8 weeks, block biopsies were histologically analyzed.

**Results:**

For the primary outcome, i.e., keratinization of the epithelium, all teeth and implants exhibited a keratinized epithelium with no histological differences among them also not in terms of statistically significant differences in length (SCTG 0.86 ± 0.92 mm, DCTG 1.13 ± 0.62 mm, and Cm, 1.44 ± 0.76 mm). Pocket formation was histologically seen at all teeth, around most implants with SCTG and DCTG, however not in the CM implant group. The connective tissue grafts showed hardly signs of degradation, whereas the CM was partly degraded and integrated in connective tissue. The mean gain in gingival height was similar in all experimental groups (SCTG 3.89 ± 0.80 mm, DCTG 4.01 ± 1.40 mm, CM 4.21 ± 0.64 mm). Statistically significant differences were found in the height of the junctional epithelium between the control teeth and the connective tissue groups (*p* = 0.009 and 0.044).

**Conclusions:**

In this animal model, the use of either a superficial or deep connective tissue graft or a collagen membrane did not seem to have any impact on the epithelial keratinization around both teeth and implants. All procedures (CAF + SCTG/DCTG/CM) resulted in a long JE that was even longer at implants.

**Clinical relevance:**

Deep/superficial palatal connective tissue graft yielded similar keratinization around teeth/implants. Given the absence of pocket formation and inflammatory processes at implants when using a CM, CAF + CM might bear potential clinical benefits.

## Introduction

Findings from a narrative review analyzing the biology and soft tissue wound healing around teeth and implants have indicated that tissue morphogenesis of the gingival, palatal, and alveolar mucosa appears to be primarily innately determined [1]. Furthermore, it also appears that the connective tissue originating from an area originally covered by keratinized epithelium and/or from the periodontal ligament possesses the potential to induce epithelial keratinization. These conclusions are in line with those made by others indicating that granulation tissue proliferating from the alveolar mucosa appears to induce the formation of a non-keratinized epithelium, whereas the one originating from the supra-alveolar connective tissue or from the periodontal ligament would lead to a keratinized epithelium [2, 3].

Based on the above-mentioned findings, connective tissue grafts (CTGs) harvested from the palate are nowadays routinely used for the treatment of soft tissue dehiscences/recessions around teeth and for increasing the width of keratinized tissue around teeth and implants. However, clinical observations indicate that in many cases when palatal CTGs are covered by a non-keratinized mucosal flap, keratinization of the epithelial cells fails to occur. These clinical observations are supported by findings from earlier studies suggesting that CTGs harvested from deep palatal connective tissue layers may not have the same potential to induce keratinization than grafts harvested from more superficial layers. In a nicely designed experiment, a thick palatal epithelial-connective tissue graft was excised and split into two thinner grafts. i.e., one immediately subepithelial and the other one closer to the bone [4]. The grafts were transplanted into contralateral areas lacking keratinized mucosa. Following a healing period of 3 months, biopsies were excised and examined by means of routine histology, immunofluorescence, and gel electrophoresis. The results showed that while the epithelial-connective tissue grafts displayed histological and biochemical characteristics of keratinized tissue (i.e., gingiva), the deep connective tissue grafts expressed features belonging to both keratinized and non-keratinized tissue. Comparable findings in humans were also reported by others indicating that palatal connective tissue grafts or free gingival grafts transplanted into areas of non-keratinized tissue may not always develop the characteristics of keratinized tissue [5–7].

Thus, it appears that CTGs harvested from the palate may not always induce keratinization at sites with originally non-keratinized epithelium, which may be explained by differences between the palatal connective tissue grafts harvested from superficial or deeper parts to induce keratinization. Interestingly, at present, it is still unknown, whether a zone of keratinized tissue may reform following complete excision (i.e., gingivectomy) of the keratinized tissues surrounding implants (i.e., excision of both free and attached mucosa). A porcine-derived bioresorbable collagen matrix (CM) has been suggested as a potential alternative to the CTG to increase the width of keratinized tissue around implants and to treat single and multiple recessions around natural teeth and implants. The available data indicate that the use of this CM may lead to an increase of keratinized mucosa around implants and [8–11], to a certain extent, to gain of keratinized tissue width when used for recession coverage at teeth [12, 13]. Whether superficial or deep CTGs induce a different degree of keratinized tissue is not known. Thus, the aim of this study is to explore to what extent differences exist between superficial (i.e., harvested from an immediately subepithelial area) and deep (i.e., harvested from an area close to the bone) parts of palatal CTGs in determining epithelial keratinization around teeth and implants completely deprived of gingiva or keratinized mucosa, respectively. We hypothesized that both superficial or deep CTGs induce similar keratinization at teeth and implants. Furthermore, it is unknown to what extent the application of a CM may replace the use of CTGs at teeth and implants.

## Materials and methods

### Surgical procedure

The study protocol was approved by the local Committee for Animal Research, University of Szeged, Hungary No 1-74-2/2015 MAB. Six Göttingen miniature pigs were used for the study. The husbandry and care of the animals before, during, and after surgery was handled at the Surgical Research Unit, University of Szeged, Hungary. The animals received standard food and water ad libitum. Animals were premedicated using ketamine (i.m. 20 mg/kg), xylazine (i.m. 2 mg/kg), atropine (i.v. 0.05 mg/kg), and midazolam (i.v. 0.5 mg/kg) to achieve the intubation status. Inhalation anesthesia was performed with isoflurane (1.0–1.5%). Fentanyl patches (5–10 mg/kg) were used for the intraoperative analgesia, and the animals received antibiotic prophylaxis for 3 days (Duplocillin LA, 12,000 U.I./kg).

**Fig. 1:**
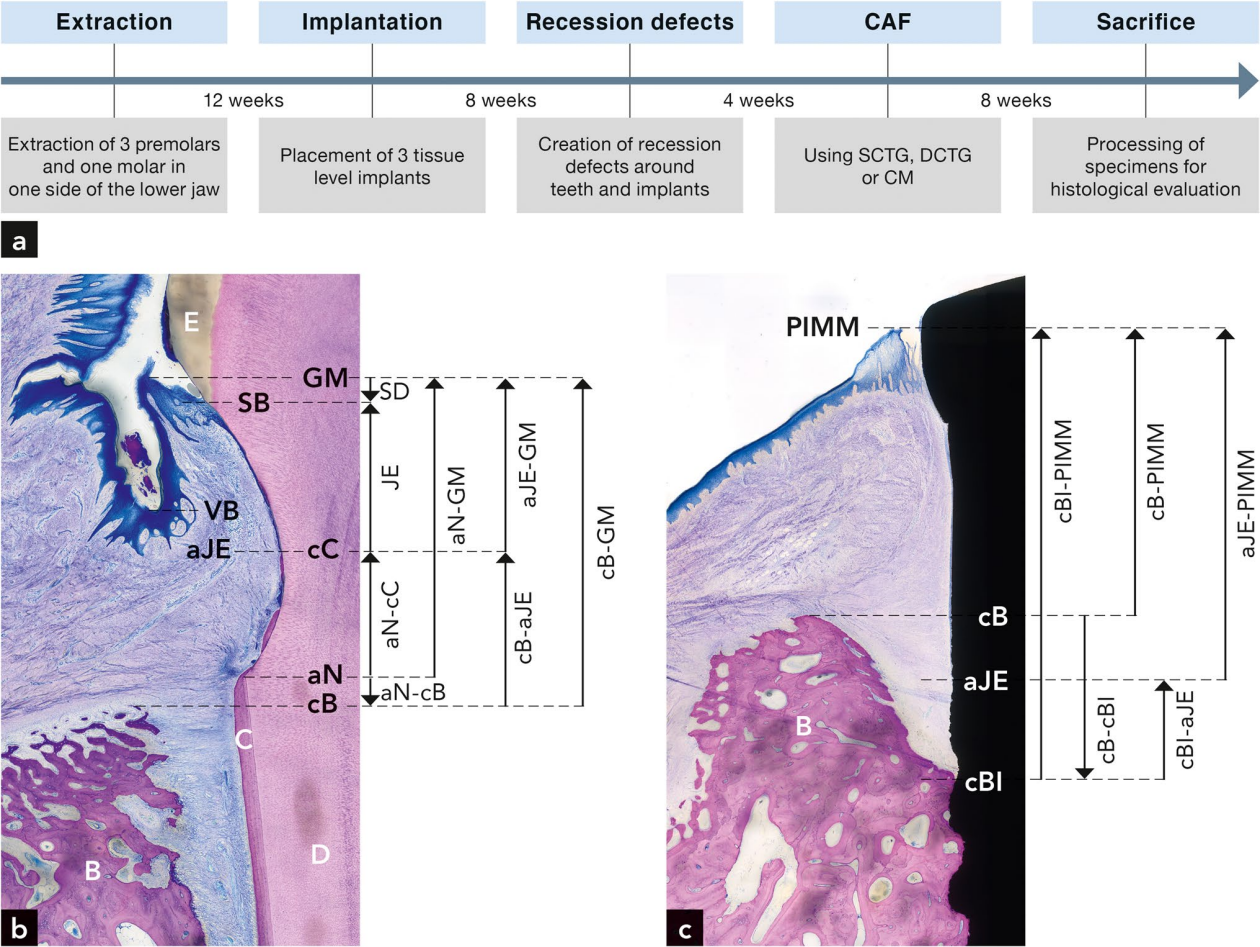
**a** Flow diagram displaying the study design with the timepoints of interventions and healing periods. **b** Landmarks around teeth for the histomorphometric measurements. GM, gingival margin; SB, sulcus bottom; VB, vestibulum bottom; aJE, the most apical extent of the junctional epithelium; cC, the most coronal extent of new cementum; aN, the most apical part of the surgically created root surface; cB, the most coronal level of bone; SD, sulcus depth; JE, junctional epithelium; aN-cC, vertical gain of new cementum; aN-cB, the most apical part of the surgically created root surface to the bone crest; aN-GM, the most apical part of the surgically created root surface to the gingival margin; aJE-GM, length of junctional epithelium plus sulcus depth; cB-aJE; the most coronal level of bone to the apical extent of the junctional epithelium; cB-GM, biologic width; c, cementum; d, dentin; b, bone. **c** Landmarks and distance measurements around implants. PIMM, peri-implant mucosal margin; aJE, the most apical extent of the junctional epithelium; cBI, the most coronal level of bone in contact with the implant; cBI-PIMM, biologic width; cB-cBI, vertical distance from the bone crest to the most coronal bone level in contact with the implant; cB-PIMM, vertical distance from the bone crest to the peri-implant mucosal margin; cBI-aJE, the most coronal level of bone in contact with the implant to the most apical extent of the junctional epithelium; aJE-PIMM, length of junctional epithelium plus sulcus depth; b, bone

The study design is summarized in Fig. 1a. In one side of the lower jaw, the second, third, and fourth premolars as well as the first molar were extracted. After 12 weeks of healing, three tissue level implants (8–10 mm long; Straumann®) were placed. After 8 weeks of healing, a soft tissue dehiscence was surgically created around the implants. Around the contralateral second, third, and fourth premolars, isolated Miller Class II recession defects were surgically created by completely removing the buccal gingiva, bone and root cementum using blades, bone chisels, and slowly rotating burs under copious rinsing with sterial saline according to previously described protocols [14, 15]. The so created defects measured about 5 mm in depth and 4 mm in width apically to the cemento-enamel junction. The exposed root and implant surfaces were left untreated for 4 weeks to allow soft tissue healing and plaque accumulation and to mimic closer a chronic recession-type defect.

After 4 weeks of healing, the defects were treated. First, the exposed parts of the roots of the teeth were meticulously cleaned with Gracey curettes (Hu-Friedy, Chicago, IL, USA); the implants received a supramucosal cleaning using rubber cups and a polishing paste (Zircate, Prophy Paste; Dentsply, Konstanz, Germany). Around teeth the most apical part of the before surgically exposed root surface was marked with a small bur (diameter 2 mm) to create a reference mark for the histometric analysis. At the implants, clinical defect height (CDH) was measured at the mid-buccal aspect from the implant shoulder (IS) to the bottom of the mucosal recession. The defects were treated using a CAF described by Allen and Miller (1989) and a CM or a CTG. Two vertical releasing incisions were placed that were 6 mm longer than the recession defects. In case a CTG was selected, the needed amount of tissue was harvested from the palate according to the technique described by Hürzeler and Weng (1999) measuring 0.5 mm less than the size of the vascular bed in mesio-distal length and 5 mm in corono-apical direction.

Using a computer-generated randomization program, the defects in each quadrant were treated as follows:

1. CAF + superficial CTG (SCTG) around teeth and implants
2. CAF + deep CTG (DCTG) around teeth and implants
3. CAF + CM (Mucograft®, Geistlich, Wolhusen, Switzerland) around teeth and implants

The flaps were closed with 6–0 monofil (Polypropylene, Stoma, Emmingen-Liptingen, Germany) suture material. Sutures were removed at 2 weeks. The animals were euthanized after 8 weeks of healing.

### Histologic processing

The lower jaws were removed and chemically fixed by immersion in 10% buffered formalin supplemented with CaCl_2_ for 3 weeks. The specimens were rinsed in running tap water, dehydrated in ascending concentrations of alcohol, and embedded in methylmethacrylate, as previously described [16, 17]. Each tooth and implant was sectioned parallel to its longitudinal axis in a bucco-lingual direction, resulting in two to three undecalcified ground sections of ~ 500 μm thickness. The sections were ground to a final thickness of 80 μm, superficially stained with toluidine blue and basic fuchsin and the two central-most sections were used for descriptive and histomorphometric analyses.

### Descriptive histology

The descriptive analysis was performed directly under the microscope. Keratinization/non-keratinization as well as presence/absence and extent of inflammation were evaluated in the sections stained with toluidine blue/fuchsin. For comparative reasons, one untreated first molar per animal served as internal control for the descriptive analysis.

### Histomorphometry

All ground sections were digitalized using a Zeiss Axio Imager.M2 microscope with an automatic scanning stage, a digital camera, and a stitching software called ZEN (Zeiss Efficient Navigation). All histometric measurements were performed at buccal sites blindly by one experienced and calibrated investigator using the ZEN software.

#### Primary outcome: keratinization of the epithelium

Measurements around teeth: The length of the keratinized tissue (from the gingival margin to the mucogingival junction), the length of the non-keratinized tissue (from the mucogingival junction to the bottom of the vestibulum), and the ratio between them were measured and calculated, respectively.

Measurements around implants: The length of keratinized tissue, length of non-keratinized tissue, and ratio of keratinized to non-keratinized tissue were planned to be measured. However, since many implants were submerged or partly submerged, these measurements were not possible.

#### Secondary outcomes

The following landmarks were identified around teeth (Fig. 1b):

- GM: gingival margin
- SB: sulcus bottom
- aJE: the most apical extent of the junctional epithelium
- cC: the most coronal extent of new cementum
- aN: the most apical part of the surgically exposed root surface, i.e., the gingival margin before flap advancement, marked with a notch
- cB: the most coronal level of bone = bone crest
- VB: the bottom of the vestibulum

The following vertical distance measurements were performed (Fig. 1b):

- aN-GM: gain in gingival height
- cB-GM: the biologic width, i.e., the vertical distance from cB to GM
- aJE-GM: length of junctional epithelium plus sulcus depth
- cC-aJE: length of connective tissue adhesion
- SB-GM: sulcus depth
- aN-cC: vertical gain of new cementum
- aN-cB: apical part of the notch to the bone crest

The following landmarks around implants were determined (Fig. 1c) according to Schwarz et al. [18]:

- PIMM: peri-implant mucosal margin
- aJE: the most apical extent of the junctional epithelium
- cBI: the most coronal level of bone in contact with the implant
- cB: the most coronal level of bone = bone crest

The following vertical distance measurements around implants were performed (Fig. 1c):

- cBI-PIMM: biologic width, i.e., vertical distance from cBI to the peri-implant mucosal margin
- cB-PIMM: vertical distance from the bone crest to the peri-implant mucosal margin
- cB-cBI: vertical distance from the bone crest to the most-coronal bone level on the implant
- aJE-PIMM: vertical length of junctional epithelium plus sulcus depth
- cBI-aJE: vertical length of soft connective tissue compartment

### Statistical analysis

Statistical analysis was performed using GraphPad Prism 9, Version 9.3.1 (GraphPad Software, Inc. CA, USA). Means and standard deviations for each histomorphometric parameter were calculated with the animal being the experimental unit for all the comparisons (*n* = 6). Due to the small sample size and the non-parametric distribution of the data, differences between groups were analyzed using the Kruskal-Wallis test followed by Mann-Whitney test with Bonferroni correction. The significance level was set at *p* < 0.05.

## Results

The healing was uneventful in all animals without wound dehiscence or other major complications. Out of 18 teeth in the three groups, one tooth was lost in vivo (group CM). Furthermore, all 6 molars, used as internal control teeth, were available for the descriptive analysis.

### Teeth

#### Descriptive histology

##### CAF + SCTG (Figs. 2a and 3a and b)

**Fig. 2:**
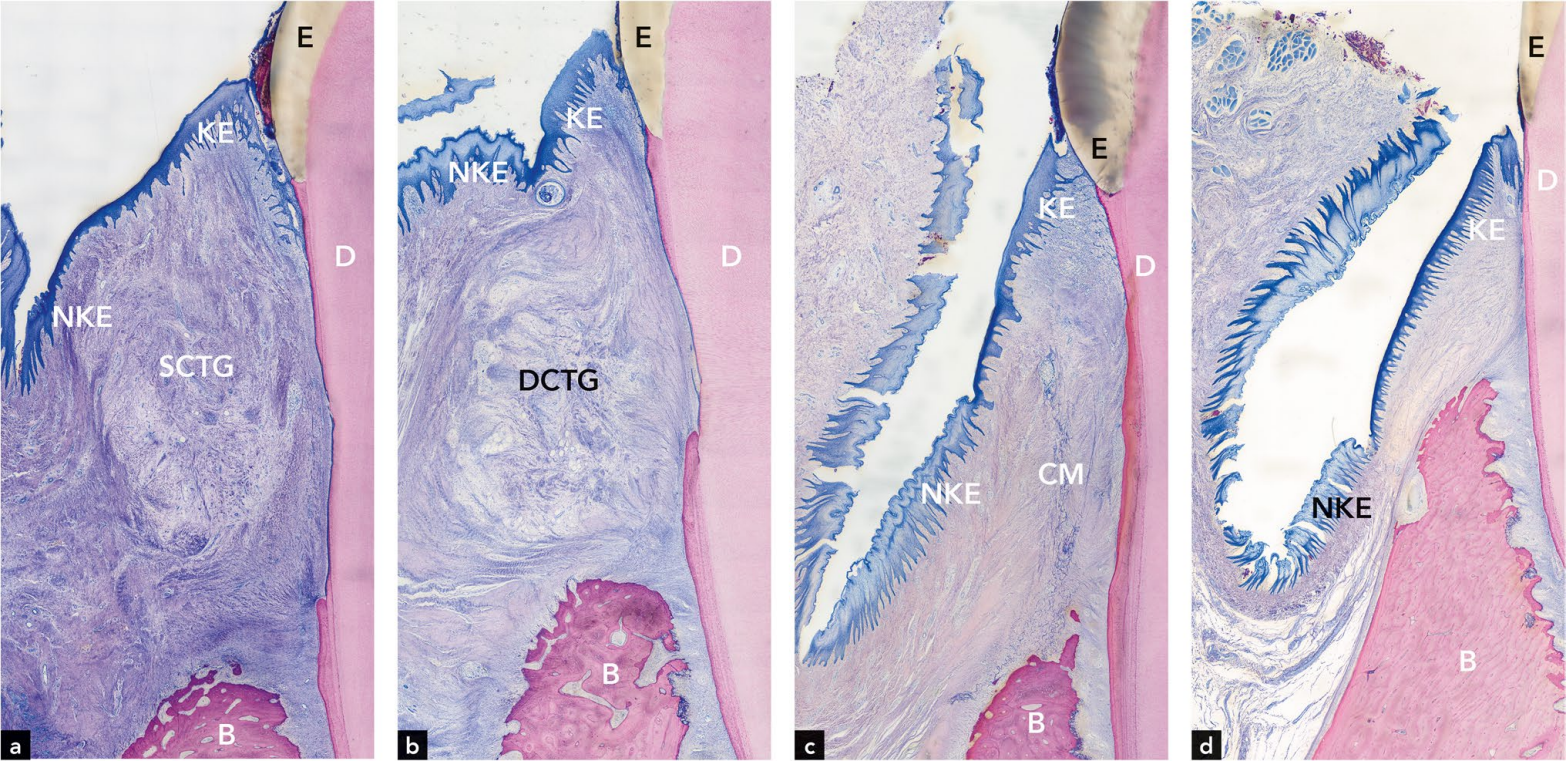
Representative micrographs illustrating the vestibulum around teeth and the encapsulated configuration of the connective tissue grafts in **a** the SCTG group, **b** the DCTG group, **c** the CM group, and **d** the control group. KE, keratinized epithelium; NKE, non-keratinized epithelium; SCTG, superficial connective tissue graft; DCTG, deep connective tissue graft; CM, collagen matrix; E, enamel; D, dentin; B, bone

All teeth had a normal keratinized oral gingival epithelium consisting of 4 strata (Fig. 2a). Owing to its volume, the SCTG appeared to widen the gingiva and to impact the spatial configuration of the vestibulum; i.e., it appeared to lift up the bottom of the vestibulum (Fig. 2a).

**Fig. 3:**
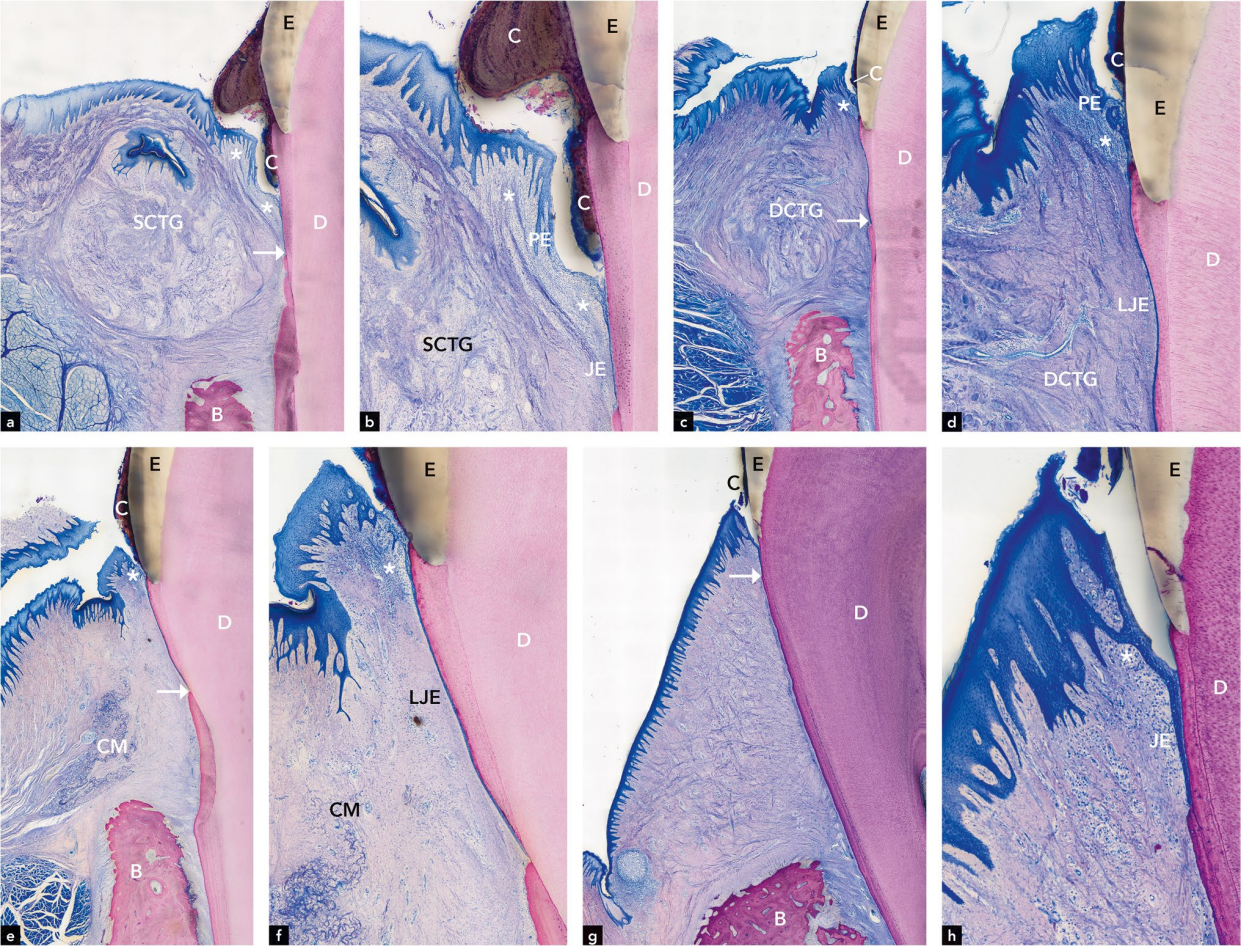
Representative micrographs illustrating the gingiva at teeth in the SCTG group **a** and **b** (b= higher magnification of **a**) the SCTG group, (**c** and **d** higher magnification of **c**) the DCTG group, (**e** and **f** higher magnification of **e**) the CM group, and (**g** and **h** higher magnification of **g**) the control group. Arrows indicate the apical end of the junctional epithelium. E, enamel; C, calculus; D, dentin; SCTG, superficial connective tissue graft; DCTG, deep connective tissue graft; CM, collagen matrix; B, bone

In all 6 teeth, the bone crest level was buccally lower than lingually. The junctional epithelium was quite long. Epithelial inclusions in the gingival connective tissue were found in 2 teeth, food impaction at 1 tooth, and multinucleated giant cells around a foreign body material at 1 tooth. The connective tissue graft was clearly discernible. Circularly, the border region did hardly show any signs of graft tissue integration into the surrounding tissue (Fig. 3a and b). Gingival pocket formation with supragingival and subgingival calculus and biofilm was found in 5 out of 6 teeth (Fig. 3b). Peri-pocket inflammation was found in all teeth with gingival pockets.

##### CAF + DCTG (Figs. 2b and 3c and d) 

All teeth had a normal, keratinized oral gingival epithelium consisting of 4 strata (Fig. 2b). Owing to its volume, DCTG appeared to impact the spatial configuration of the gingiva and the vestibulum; i.e., it appeared to widen the gingiva and lift up the bottom of the vestibulum (Fig. 2b).

In all 6 teeth, the bone crest level was buccally lower than lingually. The junctional epithelium was either long or very long. Epithelial inclusions in the gingival connective tissue were found in 2 teeth, food impaction in 1 tooth, and multinucleated giant cells around a foreign body material in 1 tooth. The connective tissue graft was clearly distinguishable from the surrounding tissue with hardly any signs of graft tissue integration into the surrounding tissue (Fig. 3c). Gingival pocket formation with subepithelial calculus and biofilm was found in all 6 teeth. Peri-pocket inflammation was also found in all teeth (Fig. 3d).

##### CAF + CM (Figs. 2c and 3e and f)

All teeth had a normal, keratinized oral gingival epithelium consisting of 4 strata (Fig. 2c). The spatial configuration of the keratinized and non-keratinized epithelium and the vestibulum were very similar to the situation around control teeth; i.e., the gingiva was thin and the bottom of the vestibulum was not elevated (Fig. 2c).

In all 5 teeth, the bone crest level was buccally lower than lingually. The junctional epithelium was either long or very long. Epithelial inclusions in the gingival connective tissue were not found. Food impaction was found in 1 tooth, a mini abscess in 1 tooth, and residual CM was found in the gingival connective tissue of all 5 teeth. The CM was partially integrated into the surrounding tissue and only remnants of the matrix could be detected (Fig. 3e and f). Gingival pocket formation, subepithelial calculus, biofilm, and peri-pocket inflammation were found in all 5 teeth (Fig. 3f).

##### Control teeth (untreated molars; Figs. 2d and 3g and h))

The oral gingival epithelium consisted of 4 strata and was keratinized (Fig. 2d). Of all groups, the keratinized epithelium of the control teeth demonstrated the most regular configuration of rete pegs (Fig. 2d).

The junctional epithelium was very short and terminated at or slightly apical to the cemento-enamel junction (Fig. 3g). All 6 teeth demonstrated a healthy gingiva with physiologically normal minimal signs of inflammation. Five teeth presented with very small gingival pockets (Fig. 3h), whereas in one tooth, massive calculus and a slightly deeper gingival pocket were found. The distance between the cemento-enamel junction and the bone crest was quite large in this tooth type, but no signs of bone resorption and pathology were observed.

### Histomorphometry

#### Epithelium

**Fig. 4:**
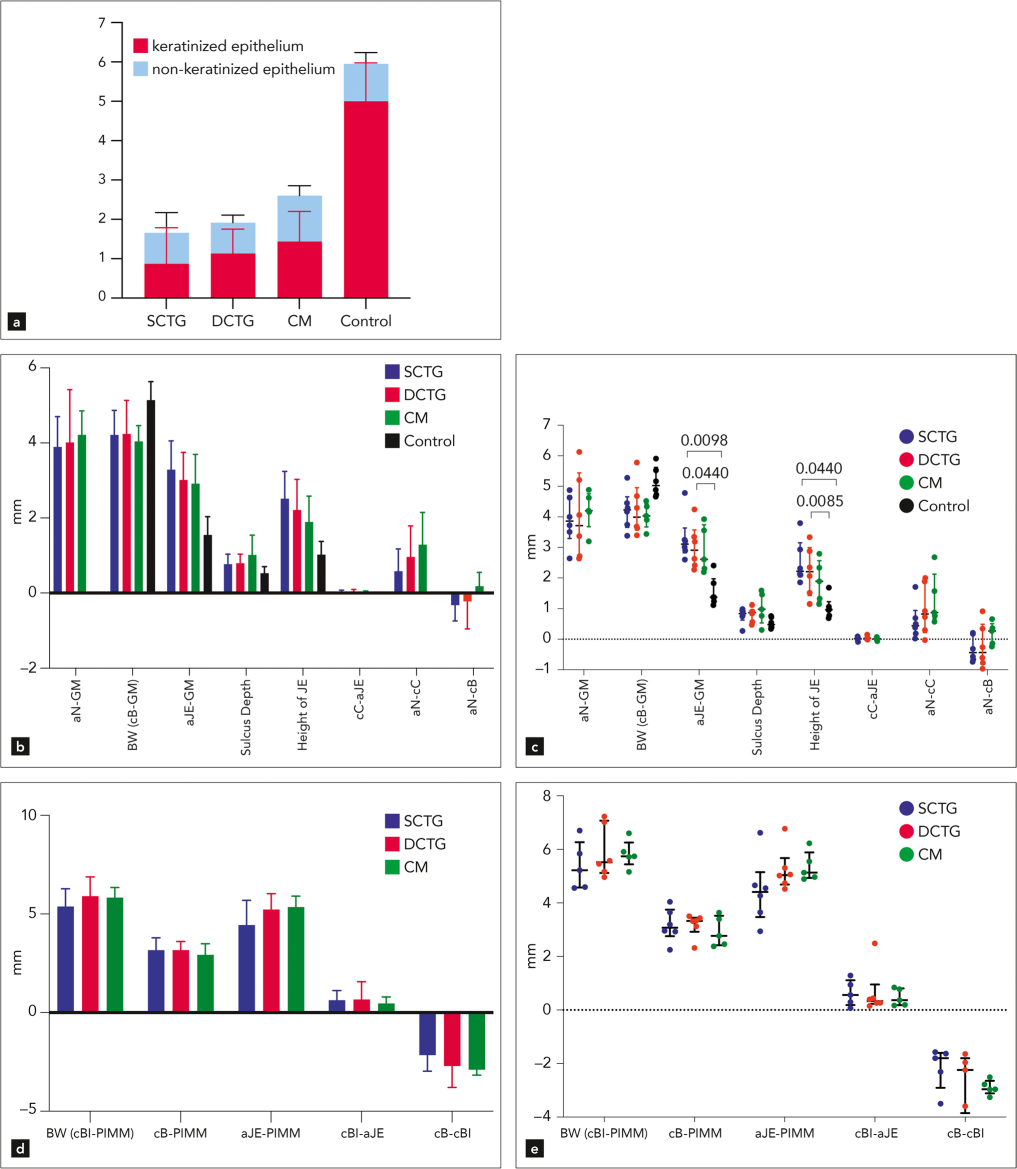
Graph representing mean and standard deviation of keratinized and non-keratinized epithelium (**a**) and of the histomorphometrically evaluated parameters around teeth (**b**). In **c**, the bars represent the median and the whiskers the interquartile range. Significance was set at *p* < 0.005. SCTG, superficial connective tissue graft; DCTG, deep connective tissue graft; CM, collagen matrix; BW(cB-GM), biologic width; aJE-GM, apical extent of the junctional epithelium – gingival margin; JE, junctional epithelium; cC-aJE; most coronal extent of new cementum — apical extent of the junctional epithelium; aN-cC, apical extent of the surgically exposed root surface — most coronal extent of new cementum; aN-cB, apical extent of the surgically exposed root surface — most coronal level of bone (bone crest). Graph illustrating the histomorphometrically evaluated parameters around implants (**d**) with means and standard deviations. In **e**, the bars represent the median and the whiskers the interquartile range. Significance was set at *p* < 0.005. SCTG, superficial connective tissue graft; DCTG, deep connective tissue graft; CM, collagen matrix; BW(cBI-PIMM), biologic width; cB-PIMM, bone crest — peri-implant mucosal margin; apical extent of the junctional epithelium — gingival margin; JE, junctional epithelium; cC-aJE; most coronal extent of new cementum — apical extent of the junctional epithelium; aN-cC, apical extent of the notch — most coronal extent of new cementum; aN-cB, apical extent of the notch — bone crest

The results of the histomorphometric analysis are presented in Table 1 and Fig. 4a. The length of the keratinized epithelium was smallest in the SCTG group. The ratio keratinized epithelium to non-keratinized epithelium was similar among all experimental groups, i.e., about 50:50, however different in the control teeth where the ratio averaged 80:20 (SCTG: 49.92 ± 23.50% to 50.07 ± 23.05%; DCTG: 56.58 ± 13.60% to 43.41 ± 13.60%; CM: 53.38 ± 9.51% to 46.61 ± 9.51%; control: 83.49 ± 6.27% to 16.50 ± 6.27%). Comparing the 3 experimental groups with each other, no statistically significant difference could be discerned between the groups in terms of keratinized epithelium length for SCTG, DCTG, and CM (0.86 ± 0.92 mm, 1.13 ± 0.62 mm, 1.44 ± 0.76 mm). Compared to the untreated control tooth group, the keratinized epithelium in both CTG groups was statistically significantly shorter (*p* = 0.0025 and *p* = 0.0228). No statistically significant difference, however, did exist between the control tooth group and the CM group (*p* = 0.1814). The length of the non-keratinized epithelium was in all experimental groups and in the control group about the same.

**Table 1:** Assessed parameters at teeth

	Keratinized epithelium in mm	Non-keratinized epithelium in mm	Whole length of epithelium in mm	aN-GM in mm	BW (cB-GM) in mm	aJE-GM in mm	SB-GM	Height of JE (mm)	cC-aJE in mm	aN-cC in mm	aN - cB (mm)
SCTG	0.8697 ± 0.9203	0.7877 ± 0.5129	1.657 ± 1.115	3.897 ± 0.8092	4.220 ± 0.6507	3.290 ± 0.7675	0.7714 ± 0.2623	2.519 ± 0.7244	0.01742 ± 0.06075	0.5806 ± 0.5988	− 0.3165 ± 0.4196
DCTG	1.136 ± 0.6201	0.7827 ± 0.1917	1.918 ± 0.6908	4.019 ± 1.407	4.245 ± 0.8891	3.014 ± 0.7376	0.7998 ± 0.2400	2.214 ± 0.8190	0.03753 ± 0.05828	0.9665 ± 0.8246	− 0.2246 ± 0.7243
CM	1.440 ± 0.7649	1.163 ± 0.2503	2.603 ± 0.9012	4.214 ± 0.6438	4.046 ± 0.4188	2.916 ± 0.7778	1.018 ± 0.5233	1.898 ± 0.6849	0.005496 ± 0.04927	1.292 ± 0.8613	0.1827 ± 0.3700
control	5.008 ± 0.9706	0.9441 ± 0.2843	5.952 ± 0.7038		5.146 ± 0.4897	1.553 ± 0.4813	0.5285 ± 0.1778	1.024 ± 0.3509			

*SCTG* superficial connective tissue graft, *DCTG* deep connective tissue graft, *CM* collagen matrix, *aN* most apical part of the surgically exposed root surface, *GM* gingival margin, *BW* biologic width, *cB* most coronal level of bone, *aJE* most apical extent of the junctional epithelium, *SB* sulcus bottom, *cC* most coronal extent of new cementum

#### Vertical measurements

The results of the vertical measurements are presented in Table 1 and Fig. 4b and c. The gain in gingival height (aN-GM) was similar for all 3 experimental groups (3.89 ± 0.80 mm for SCTG, 4.01 ± 1.40 mm for DCTG, and 4.21 ± 0.64 mm for CM). The biologic width (BW; cB-GM) was highest at the control teeth (5.14 ± 0.48 mm) where the crestal bone was located far apical to the CEJ. Not statistically significantly, but slightly lower BW values were measured for the experimental groups (4.22 ± 0.65 mm for SCTG, 4.24 ± 0.88 mm for DCTG, 4.04 ± 0.41 mm for CM). The biologic width comprised the epithelial attachment (the junctional epithelium JE plus sulcus depth), the connective tissue adhesion (cC-aJE), the gain of new cementum (aN-cC), and the distance aN-cB. Of all assessed parameters, only two (aJE-GM and the height of JE) reached a statistically significant difference between the control and the experimental groups. However, no statistically significant difference was observed between the experimental groups for any of the assessed parameters. The distance aJE-GM was smallest in the control group, while all experimental groups had a rather long JE including the sulcus depth (SCTG: 3.29 ± 0.76 mm, DCTG: 3.01 ± 0.73 mm, CM: 2.91 ± 0.77 mm, control: 1.55 ± 0.48 mm) reaching statistical significance only for the difference between control and each of the CTG groups (*p* = 0.009 and *p* = 0.044). The same was true for the height of the JE. The sulcus depth was smallest in the control group, while the other groups all showed a much greater sulcus depth associated with slight inflammation and pocket formation. The connective tissue adhesion (cC-aJE) was extremely small in all test groups, indicating that new cementum and the apical end of the JE were either confluent or in close proximity to each other. The mean vertical gain of new cementum (aN-cC) was highest in the CM group, followed by the SCTG and the DCTG groups. Of note, the distance between the apical end of the notch (i.e., former level of the gingival margin) to the bone crest reached a positive value in the CM group, whereas this distance was negative in the SCTG and DCTG groups. This implies that vertical bone growth was clearly greater in the CM group compared to the two CTG groups.

### Implants

#### Descriptive histology

##### CAF + SCTG (Fig. 5a, b, c, d)

**Fig. 5:**
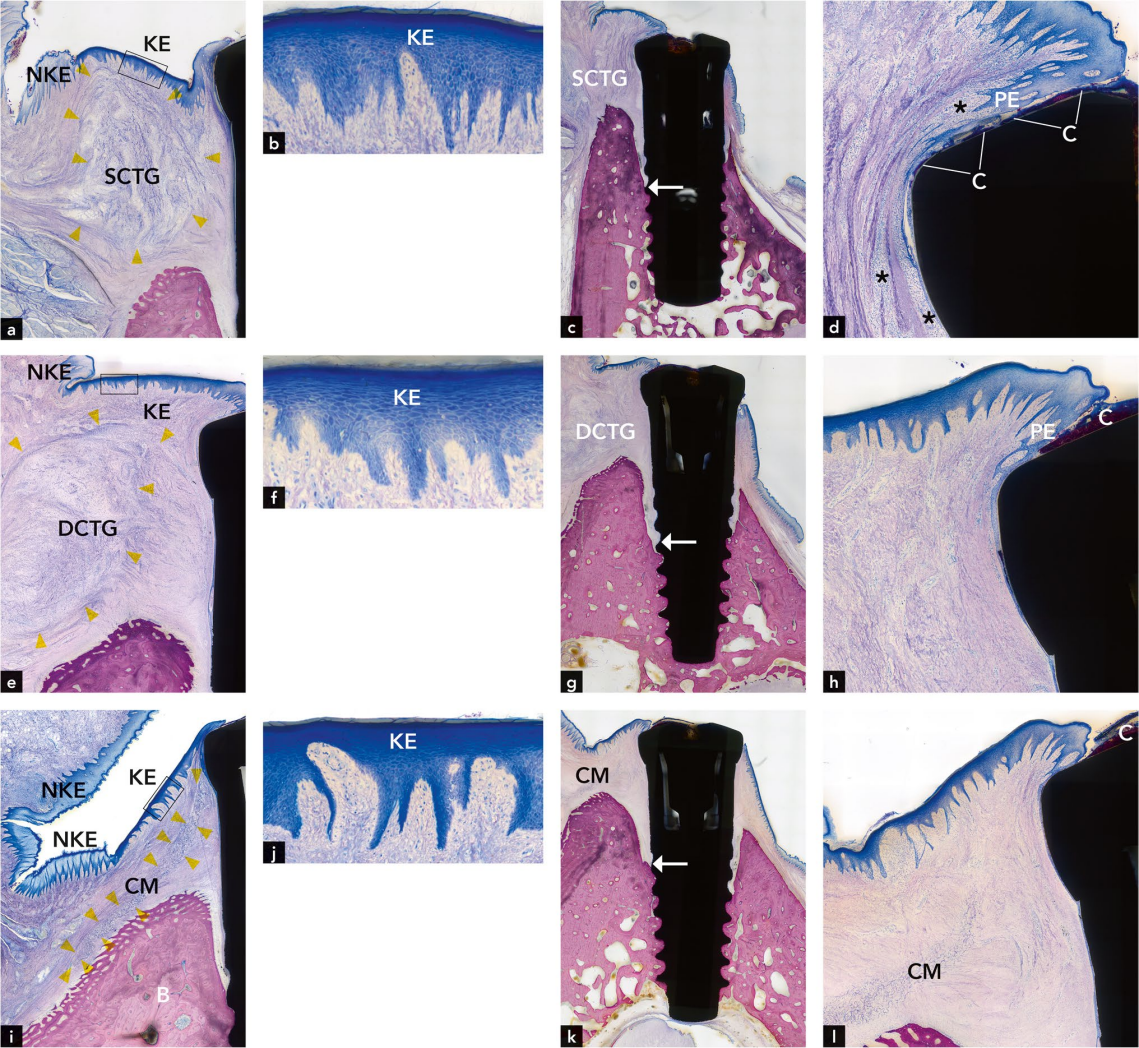
Representative micrographs illustrating the grafting area at the 3 experimental groups at implants (**a**, **e**, **i**). **b** shows the marked region in **a**, **f** in **e**, and **j** in **i** of the keratinized epithelium in higher magnification. Representative micrographs illustrating the 3 experimental groups at implants in overview (**c**, **g**, **k**) and in higher magnification of the peri-implant mucosal margin (**d**, **h**, **l**). SCTG, superficial connective tissue graft; DCTG, deep connective tissue graft; CM, collagen matrix; KE, keratinized epithelium; PE, pocket epithelium; C, calculus

The epithelium of the peri-implant mucosa facing the graft resembled a keratinized epithelium (Fig. 5a and b). There was a layer of soft connective tissue between the epithelium and the SCTG.

All 6 implants were non-submerged and demonstrated saucer-shaped bone defects both buccally and lingually (Fig. 5c). In 1 implant, advanced bone loss had occurred. Around another implant, dentin and cementum remnants were found. Small pocket formation, calculus, biofilm, and mild inflammation were observed in 4 implants (Fig. 5d). The vertical distance between the peri-implant mucosal margin and the most coronal level of the bone was conspicuously long. The junctional epithelium was long or very long, and its apical termination was always below the bone crest (Fig. 5c). The SCTG was found around all implants. It was big, round-shaped, and its localization in relation to the keratinized epithelium varied between implants (Fig. 5a).

##### CAF + DCTG (Fig. 5e, f, g, h)

The epithelium of the peri-implant mucosa facing the graft resembled a keratinized epithelium (Fig. 5e and f). All 6 implants were non-submerged and demonstrated saucer-shaped bone defects both buccally and lingually (Fig. 5g). In 1 implant, advanced bone loss had occurred. Small pocket formation, calculus, biofilm, and mild inflammation were observed in 4 implants (Fig. 5h). The vertical distance between the peri-implant mucosal margin and the most coronal level of the bone was conspicuously long, and the junctional epithelium was very long and its apical termination always below the bone crest (Fig. 5g). The DCTG was found around all implants, was big, round-shaped, and its localization in relation to the keratinized epithelium varied between implants (Fig. 5e). A layer of soft connective tissue was interposed between the epithelium and the DCTG.

##### CAF + CM (Fig. 5i, j, k, l)

The epithelium of the peri-implant mucosa facing the coronally located CM resembled a keratinized epithelium (Fig. 5i and j). One implant was lost in situ. Out of 5 implants, 2 implants were submerged, whereas 3 implants were non-submerged. Around all implants, saucer-shaped bone defects were observed both buccally and lingually (Fig. 5k). One implant showed a very small pocket formation. All other implants had no pocket formation (Fig. 5l). Healthy peri-implant soft tissue conditions with minimal (physiologically normal) inflammation were observed around all implants. Around all implants, the most coronal level of bone in contact with the implant (cBI) was located very apically (Fig. 5k). Likewise, the vertical distance between the bone crest and the most coronal bone in contact with the implant was conspicuously long. The junctional epithelium was very long and its apical termination always below the bone crest (Fig. 5k). Residual CM was present in the soft connective tissue around all implants (Fig. 5i). It was thin and elongated and its localization in relation to the keratinized epithelium varied between implants. There was mostly a thick layer of connective tissue between the epithelium and the CM.

Although all implants were surrounded by a collar of keratinized mucosa (Fig. 5a, e, i), its length could not be determined histomorphometrically, since not all implants showed transmucosal healing and most implant healing caps were partially overgrown by peri-implant mucosa.

### Histomorphometry

The histomorphometric data of the implants are shown in Table 2 and Fig. 4d and e. For none of the parameters, a statistically significant difference among the groups was achieved. The biologic width (cBI-PIMM) comprised of cBI-aJE and aJE-PIMM was very similar in all three groups. Likewise, no significant differences were seen for the distance between the bone crest and the peri-implant mucosal margin (cB-PIMM). The distance between the apical end of the junctional epithelium and the peri-implant mucosa (aJE-PIMM), which corresponds to the height of the junctional epithelium plus the sulcus depth, varied from 4.44 ± 1.24 mm to 5.35 ± 0.55 mm and was considerably longer around implants than around corresponding teeth. The distance between bone on the implant and the apical end of the junctional epithelium (cBI-aJE), corresponding to the connective tissue adhesion on the implant, was short in all 3 groups. The height of the saucer-shaped bone deficiency (cB-cBI) was greatest in the.

**Table 2:** Assessed parameters at implants

	BW(cBI-PIMM) in mm	cB-PIMM in mm	aJE-PIMM In mm	cBI-aJE in mm	cB-CBI mean ± SD
SCTG	5.38 ± 0.90	3.17 ± 0.62	4.44 ± 1.24	0.63 ± 0.48	− 2.16 ± 0.80
DCTG	5.90 ± 0.97	3.17 ± 0.43	5.23 ± 0.79	0.66 ± 0.89	− 2.68 ± 1.04
CM	5.82 ± 0.51	2.93 ± 0.56	5.35 ± 0.55	0.47 ± 0.32	− 2.89 ± 0.27

*SCTG* superficial connective tissue graft, *DCTG* deep connective tissue graft, *CM* collagen matrix;, *BW* biologic width, *cBI* most coronal level of bone in contact with the implant, *PIMM* peri-implant mucosal margin, *cB* the most coronal level of the bone (crest), *aJE* most apical part of the junctional epithelium

CM group followed by DCTG and SCTG, albeit without statistical significance.

## Discussion

This animal study investigated the healing characteristics around teeth and implants after recession coverage using either a superficial or deep connective tissue graft from the palate or a collagen matrix. We applied descriptive histological and histomorphometrical analyses to evaluate whether differences among the groups exist regarding the healing pattern, epithelial keratinization, and dimensions of soft and hard tissues around teeth and implants.

In terms of keratinization, all groups demonstrated the formation of keratinized epithelium around both teeth and implants. In teeth, the 3 experimental groups obtained similar lengths of the keratinized epithelium, albeit significantly shorter compared to the group with the control teeth. The length of the non-keratinized epithelium was similar for the control and experimental groups. These results imply that the difference of the keratinized epithelium between control and experimental teeth might be strongly influenced by the recession defect that was surgically created. The length of the keratinized tissue around implants could not be determined due to the fact that not all implants demonstrated complete transmucosal healing and thus not equal healing conditions.

Also, in the minipig model, other studies evaluated the amount of keratinized tissue in response to treatment of gingival recession defects. CAF alone yielded about 1 mm greater width of keratinized tissue compared to CAF + CM [15]. The amount of keratinized tissue averaged 2.66 ± 0.42 mm before CAF + CTG and 3.83 ± 0.47 mm 12 weeks afterwards [14]. In our study, the keratinized epithelium at the experimental teeth measured 0.86 ± 0.92 mm (SCTG), 1.13 ± 0.62 mm (DCTG), and 1.44 ± 0.76 mm (CM). This might be partly due to differences in the histometric evaluation and to the fact that no baseline measurements (i.e., before CAF preparation) of the keratinized epithelium were taken; instead, the values after 8 weeks were compared with a control tooth. Furthermore, in the present study, only mandibular teeth and sites for implant installation were used, whereas the other studies used both maxillary and mandibular sites [14, 15].

The observation that CAF + CTG and CAF + CM resulted in an equivalent amount of keratinized tissue gain is in agreement with clinical studies [19, 20] where keratinized tissue gain averaged 1.26 mm for CAF + CTG and 1.34 mm for CAF + CM [12, 21].

The present study has failed to show that superficial and deep connective tissues display different inherent characteristics to induce keratinization at the recipient site as was suggested by Ouhayoun et al. (1988). However, when interpreting the here presented results, it must be kept in mind that the connective tissue grafts were covered with a rather thick layer of flap which might have hindered the direct influence of cells within the grafts onto the epithelium. Indeed, the results of Ouhayoun et al. (1988) showed that deep connective tissue grafts had not the same ability to induce keratinization as connective tissue grafts that were harvested closer to the epithelium [4]. A recent review with meta-analysis corroborated the superior outcome of superficial grafts, reporting a mean recession coverage of 89.3% for deeper connective tissue grafts and 94.0% for de-epithelialized superficial connective tissue grafts (Travelli et al., 2019). In terms of keratinized tissue gain and recession reduction, better results were found in favor of the superficial graft [22].

Whether inflammatory processes may affect tissue keratinization is still a matter of discussion. Chronic or acute inflammation, experimentally induced in animals, was not able to convert tissue keratinization [23, 24]. On the other hand, a reduction of gingival inflammation allowed sulcular keratinization to occur [25]. In the present study, pocket formation with subgingival calculus formation and inflammatory processes were observed at nearly all (experimental) teeth and around the implants receiving a CTG. In contrast, the implants that received a CM showed no pocket formation and healthy peri-implant soft tissue conditions with minimal (physiologically normal) inflammation. Nevertheless, no difference was observed among inflamed and non-inflamed conditions in terms of epithelial keratinization. One possible explanation for the difference in pocket formation between the CM and the CTG groups at the implants is that the rather voluminous, spherical CTGs substantially lifted the bottom of the vestibulum and may have hampered tight sealing between flap and teeth/implants thus favoring plaque-induced inflammation. Conversely, the less voluminous and rather flat CMs did not result in an elevation of the bottom of the vestibulum and around implants allowed for a undisturbed healing.

One interesting finding was that after 8 weeks of healing, both superficial and deep connective tissue grafts hardly showed signs of degeneration or integration into the surrounding tissues. This observation was made for both teeth and implants. So far, little is known about the temporal sequence of tissue degradation/integration of transplanted connective tissue grafts from the palate. The seminal studies of Karring et al. (1971) in monkeys not only first addressed the question of the specificity of the epithelium but also described healing from a few days up to 12 months [26]. After 3 months of healing, the transposed tissues had partly degenerated [27, 28]. But here it has to be kept in mind that the surgical techniques and species differed in the latter and the present study.

In the present study, all experimental groups yielded similar results in terms of biologic width. Of note, at control teeth the BW averaged 5.1 mm which is considerably higher than in other species or in humans [29]. In the SCTG and DCTG groups around teeth, the JE measured 2.51 ± 0.72 mm and 2.21 ± 0.81 mm, what is significantly longer than at control teeth. These results strongly suggest that the surgical manipulation of the soft tissue resulted in a repair process with an apical migration of the JE. Nevertheless, these results are comparable with previous findings in dogs [30] and minipigs, where treatment with CAF alone resulted in 2.79 ± 0.77 mm and CAF + CM in 2.26 ± 0.23 mm of JE [15]. At the implants, the JE was even longer. Also, at the implants, the distance cB-PIMM averaged 3.17 ± 0.62 mm and 3.17 ± 0.43 mm for SCTG and DCTG, while a bit less for CM. Here, it might be possible that the connective tissue grafts may induce some kind of bone resorption likewise to root resorptions that have rarely been described [31–33].

Much can be discussed about the limitations of this model. The miniature pig model might not be perfectly suitable for this research question considering that it displays a different and for this type of surgical procedure more challenging anatomy of the vestibulum compared to humans. Other researchers have performed coronally advanced flap surgeries after connective tissue or biomaterial transplantations in the minipig in both the mandible and maxilla [14, 15] or in a more anterior position [15]. Consequently, the 3 experimental groups resulted in a deep (CM group), very shallow, missing, or directly rising vestibulum (CTG groups). The thickness of the transplanted materials together with the anatomy at these sites may account for the differences between CM and the two CTG groups. Furthermore, harvesting superficial and deep connective tissue from the palate is difficult to standardize. Implant placement and positioning in relation to hard and soft tissues had to be adapted to the anatomical situation and do not fully correspond to the situation in humans which might have been one reason for the saucer-shaped defects to occur. Furthermore, some of the implants resulted in a submerged or semi-submerged healing, while few healed fully transmucosally. During healing, adequate measures of plaque control and postoperative care were not feasible in this animal model. Consequently, tissues around all the teeth and most of the implants showed signs of inflammation and calculus formation on teeth and implants. Horizontal measurements along any level for both teeth and implants were not doable for all samples. The control teeth were not planned but then included in order to have a comparison with normal histomorphometric parameters around teeth (i.e., JE, soft connective tissue height, bone level). However, while the control teeth were molars, all experimental teeth were premolars and thus not fully comparable. Finally, a rather small number of teeth and implants were treated by two surgeons. This might have caused some inter-operator variation.

To better understand the characteristics and effects of superficial and deep connective tissue grafts, further studies and more suitable models are warranted.

## Conclusion

Around both teeth and implants, CAF + SCTG/DCTG/CM resulted in the formation of keratinized epithelium with no differences between SCTG and DCTG. The length of the keratinized epithelium was conspicuously shorter at the experimental teeth compared to the control teeth. All experimental teeth and implants receiving SCTG or DCTG showed pocket formation with subgingival calculus and inflammation, whereas implants receiving CM displayed healthy peri-implant soft tissue conditions what implies that CAF + CM was superior to CAF + SCTG/DCTG regarding this aspect. All procedures (CAF + SCTG/DCTG/CM) resulted in a long JE that was even longer at the implants. After 8 weeks of healing, both SCTG and DCTG hardly showed any signs of degeneration or integration into the surrounding tissues.

## Data Availability

Data are available on request.
